# Ultrasound in endometrial cancer: evaluating the impact of pre-surgical staging

**DOI:** 10.3389/or.2025.1446850

**Published:** 2025-03-05

**Authors:** Mariana Rei, João Francisco Bernardes, Antónia Costa

**Affiliations:** ^1^ IPO-Porto Research Centre, Portuguese Oncology Institute, Porto, Portugal; ^2^ Department of Gynecology-Obstetrics and Pediatrics, Faculty of Medicine, University of Porto, Porto, Portugal; ^3^ Department of Obstetrics and Gynecology, Centro Hospitalar Universitário de São João, Porto, Portugal

**Keywords:** endometrial neoplasia, uterine cancer, pelvic ultrasound, myometrial invasion, cervical invasion, staging and surgical approach

## Abstract

Preoperative staging in endometrial cancer has recently been implied as an important factor in accurately selecting low-risk cases, ultimately avoiding unnecessary lymph node debulking. Transvaginal ultrasound seems promising in clinical staging as it offers the possibility to assess the depth of myometrial infiltration and cervical stromal invasion. This commonly available, non-invasive, and low-cost modality serves as an accurate alternative to MRI, especially in middle- and low-income countries, where MRI may not be promptly available and cost is an important issue. This review aims to summarize the progressive role of clinical implementation of pelvic ultrasonography in the locoregional staging of endometrial carcinoma and to compare its accuracy with other preoperative methods.

## 1 Introduction

Endometrial carcinoma (EC) is the sixth most common malignancy diagnosed in women and the most common gynecological cancer in high-income countries, with 417,000 new diagnosis globally in 2020 (1). Over the last 30 years, the overall incidence of EC has increased by 132%, reflecting a progressive upsurge in the prevalence of risk factors such as population aging, a reduction in benign hysterectomies, and the increasing prevalence of obesity, with the latter being the major underlying cause (2). This trend is mostly noticed in high-income countries, with the highest rate in North America (86.6/100.000), followed by east and central Europe (52.5 - 21.9/100.000) (2). However, an increase in the age-standardized incidence rate has been reported globally, including in Sub-Saharan Africa (3).

EC usually presents at an early stage with postmenopausal bleeding, and most cases are diagnosed at FIGO (International Federation of Gynecology and Obstetrics) stage I, with a 5-year survival rate of 90% (4).

The histologic diagnosis is usually based upon the results of an endometrial biopsy or hysterectomy specimens. Pelvic ultrasonography and endometrial sampling have shown efficacy in the initial assessment of postmenopausal women with uterine bleeding (5, 6).

The most important prognostic features for EC are FIGO stage, myometrial infiltration, histological type, and differentiation grade (7). Lately, molecular and genetic findings, since the publication of The Cancer Genome Atlas (TCGA) data, have shed light on the diverse biological nature of this group of endometrial cancers and their differing prognostic outcomes (8). Prognosis is poorer in women with high-risk EC, as defined by the presence of deep myometrial invasion, cervical stromal invasion, grade 3 tumors, or non-endometrioid histotypes, leading to an increased risk of lymph node metastasis. In contrast, patients with low-risk cancer do not benefit from systematic pelvic and para-aortic lymph node dissection as it does not improve survival and may lead to increased surgical morbidity (9, 10). Even in low-grade endometrioid tumors, the risk of lymph node metastasis increases from 4% to 15% if the tumor invades the outer half of the myometrium (11). Molecular profiling within the high-grade endometrioid group can distinguish an excellent prognosis group (POLEmut in early-stage disease) from a notably poorer prognosis group (p53 abnormal [p53abn]) (8).

Classically, EC is a surgically staged disease (8). For years, intraoperative frozen sectioning for the evaluation of myometrial and cervical infiltration in clinical stage I endometrial carcinoma has been advocated in order to support the decision to proceed with lymph node staging, reporting a high accuracy for determining myometrial invasion (12) and cervical stromal invasion (13). Limitations to this approach include its time-consuming nature and the lack of expertise and reproducibility (14, 15).

Therefore, a preoperative evaluation appears to be beneficial, allowing a more carefully planned treatment, an effective selection of high-risk cases elective for more radical surgery and meanwhile obviating overtreatment in low-risk cases, as well as avoiding longer operative times due to frozen section.

## 2 Oncological staging of endometrial carcinoma

### 2.1 Surgery and lymph node status

EC is surgically staged and requires histological confirmation of type, grade, and local extent of disease as fundamental criteria for staging (8). Such criteria are established by the FIGO (8) and tumor–node–metastasis (TNM)-based Union for International Cancer Control (UICC) (16, 17).

The surgical mainstay of treatment includes total hysterectomy and salpingo-oophorectomy (15).

Lymph node status, as part of surgical staging, assumes an important role in defining prognosis and, therefore, in guiding decisions for adjuvant treatments. Pathologic and molecular criteria, lately structured into risk categories, have been used to predict lymph node metastasis and guide surgery (15). However, significant perioperative morbidity associated with pelvic and para-aortic lymph node dissection must be taken into account.

Moreover, the therapeutic role of systematic lymphadenectomy has been challenged in two randomized controlled trials, which did not report a survival benefit (9, 10, 18).

Given the morbidity of systematic lymphadenectomy and balancing the potential risk of lymph node metastasis, it is clear that a subset of patients may not benefit from systematic lymphadenectomy, especially if the risk of lymph node disease is low. Although in stage I disease, 3%–5% of women with well-differentiated tumors and superficial myometrial invasion will have lymph-node involvement, this proportion increases to 20% in poorly differentiated tumors and deep myometrial invasion (10).

Sentinel lymph node biopsy (SLNB) has been recently introduced as a reliable alternative to routine pelvic and para-aortic lymph node dissection and can be considered for staging purposes in patients with low- and intermediate-risk diseases (15). The emergence of this technique simultaneously allows the following: 1) a more intensive pathologic assessment of lymph node status with ultra-staging, which could be missed by standard evaluation (19), and 2) a substantially lower risk of postoperative morbidity related to extensive lymph node dissection (20).

The National Comprehensive Cancer Network (NCCN) panel recommends that nodal evaluation be performed in patients with EC, including para-aortic lymphadenectomy in high-risk patients (21). SLNB mapping is the preferred alternative to full lymphadenectomy in the setting of apparent uterine-confined disease (21).

An open-label, non-inferiority randomized trial (ALICE trial) aims to confirm that SLNB without systematic node dissection does not negatively impact oncological outcomes; this trial is currently completing accrual and will provide more evidence on this subject (22).

### 2.2 Preoperative work-up

Recently, molecular studies have obtained promising results in providing important information for prognosis and for predicting the response to novel therapies. The TCGA network identified four major clinically significant molecular subtypes with differing clinical prognosis: POLE-mutated (DNA polymerase epsilon), microsatellite instability high (MSI-H), copy number low, and copy number high (associated with abnormal p53 expression/TP53 mutation) (23). These studies can be performed on either biopsy or tumor surgical specimens and may impact the need for adjuvant treatments.

Concerning preoperative imaging staging, recent international guidelines include expert transvaginal or transrectal ultrasound (US) or pelvic MRI as a preoperative mandatory work-up for the management of patients with EC (15). These imaging techniques are advocated as a routine procedure in the preoperative assessment of EC in order to estimate tumor local extension and identify patients with endocervical cancer or synchronous ovarian cancer.

## 3 Methodologies for pre- and intraoperative staging: transvaginal ultrasound, pelvic MRI and frozen section

### 3.1 Transvaginal ultrasound (TVUS)—general concept

Ultrasound is usually the first examination performed in women with a history of abnormal uterine bleeding. Although there is some controversy regarding the most adequate cut-off value for endometrial thickness in postmenopausal women, most authors consider 5 mm to be the upper limit of normality. This cut-off value displays a sensitivity of 96% and a specificity of 61% in the diagnosis of EC in postmenopausal women with abnormal uterine bleeding (24).

Studies have largely assessed whether morphological features and vascular patterns on Doppler US can improve the diagnostic accuracy of EC. The International Endometrial Tumor Analysis (IETA) group in 2010 published a consensus on terminology, definitions, and measurements of the endometrium (25). The most commonly reported features of EC diagnosis, while applying the IETA terminology, include heterogeneous echogenicity, irregular or undefined endometrial–myometrial junction and multiple multifocal vessel patterns, and a moderate or high color score on Doppler evaluation (26).

### 3.2 TVUS—staging role in EC

Several studies have been evaluating the role of TVUS in clinical staging as it offers the possibility to assess the depth of myometrial infiltration and cervical stromal invasion (27). Mascilini et al. compared subjective and objective assessments of these features, concluding that subjective evaluation performs at least as well as the objective measurement techniques (28). Alcazar et al. used three-dimensional (3D) virtual navigation to determine the myometrial infiltration with high sensitivity (29). Clinical and US-based models have been proposed to predict EC, presenting a high area under the receiver-operating characteristics curve (AUC) and acceptable intra-and inter-observer agreement (30). Further studies are needed to evaluate the suggested cut-offs, the predictive and prognostic value of each imaging feature and scoring systems, and the reproducibility of objective measurement techniques.

### 3.3 US techniques for the evaluation of myometrial infiltration

A systematic review and meta-analysis reviewed the diagnostic accuracy of TVUS in the preoperative detection of deep myometrial infiltration (DMI) in patients with EC, comparing subjective and objective methods based on the full text of 24 articles, reporting on 2,773 patients between 1994 and 2014 (31). Overall pooled sensitivity, specificity, positive likelihood ratio, and negative likelihood ratio of TVUS for detecting deep myometrial infiltration were 82% (95% CI, 76%–87%), 81% (95% CI, 76%–85%), 4.3 (95% CI, 3.6–5.3), and 0.22 (95% CI, 0.16–0.30), respectively. The objective techniques applied for determining the myometrial invasion were Gordon’s approach (ratio of the distance between endometrium–myometrium interface and maximum tumor depth to the total myometrial thickness) and Karlsson’s approach (endometrial tumor thickness/anteroposterior uterine diameter ratio) (32, 33). This meta-analysis did not observe significant differences in diagnostic performance among the three methods, yet significant heterogeneity between studies was found (I^2^ range, 60.6–95.0). A potentially low reproducibility of the methods is a reasonable explanation for this high heterogeneity. Among test accuracy studies, the threshold effect was one of the primary causes of heterogeneity as different cut-offs were applied in different studies. Yet, the main limitation pointed out by the authors was that very few studies compared different approaches in the same set of patients (31).

In a prospective multicenter study including 144 women with EC, (28) aimed to compare the diagnostic accuracy of subjective ultrasound assessment with that of objective measurement techniques in the evaluation of myometrial and cervical invasion. Subjective evaluation of myometrial and cervical invasion was performed, and the following objective measurements were assessed: endometrial thickness, tumor/uterine anteroposterior (AP) diameter ratio, minimal tumor-free margin, minimal tumor-free margin/uterine AP diameter ratio, tumor volume (three-dimensional (3D)), and tumor/uterine volume (3D) ratio.

Tumor/uterine AP diameter (at cut-off, 0.53) demonstrated the best performance among all objective measurement techniques, with a sensitivity and specificity of 72% and 76%, respectively. These results did not significantly differ from those of subjective evaluation (sensitivity, 77% (P = 0.44); specificity, 81% (P = 0.32)) for the prediction of deep myometrial invasion (28).

A prospective study evaluating 169 consecutive women with well- or moderately differentiated endometrioid-type EC aimed to compare the diagnostic performance of six different ultrasound approaches in assessing myometrial infiltration (34). Approaches for assessing myometrial infiltration included the following: 1) the subjective impression of the examiner; 2) Karlsson’s criteria; 3) endometrial thickness, using a cut-off of ≥18 mm for predicting ≥50% myometrial infiltration, as suggested by (28); 4) tumor/uterine volume ratio; 5) shortest tumor distance to serosa (TDS), using a cut-off of <9 mm for predicting ≥50% myometrial infiltration; 6) Van Holsbeke’s subjective model and a cut-off of an estimated probability of ≥0.50 for predicting ≥50% myometrial infiltration (35). The subjective impression of the examiner and subjective model performed similarly, displaying sensitivity values of 79.5% and 80.5% and specificity values of 89.6% and 90.3%, respectively. Both performed significantly better than Karlsson’s criteria (sensitivity 31.8%, *p* < 0.05) and endometrial thickness (sensitivity 47.7%, **p** < 0.05), as well as tumor/uterine volume ratio (specificity 28.3%, *p* < 0.05) and TDS (specificity 41.5%, *p* < 0.05) (34).

Frühauf et al. evaluated 210 patients with histologically proven EC in a prospective study, aiming to compare the diagnostic accuracy of subjective versus objective techniques, the last one including Karlsson’s and Gordon’s ratios (36). Subjective assessment was confirmed to be the most reliable method, showing sensitivity, specificity, and overall accuracy values of 79.3%, 73.2%, and 75.7%, respectively. Gordon’s ratio at a cut-off of 0.5 reached 69.6% sensitivity, 65.9% specificity, and 67.3% overall accuracy, while Karlsson’s ratio with the same cut-off reached 56.3% sensitivity, 76.4% specificity, and 68.1% overall accuracy (36).

In 2020, Verbakel et al. compared the performance of US measurements and subjective US assessment in detecting deep myometrial invasion (MI) and cervical stromal invasion (CSI) in women with EC, overall and according to whether they had low- or high-grade disease separately, comprising 1,538 patients from the IETA-4 prospective multicenter study (37). The sensitivity and specificity of subjective assessment for detecting deep MI were 70% and 80%, respectively, in patients with a Grade-1 or -2 endometrioid or mucinous tumor, compared to 76% and 64% in patients with a Grade-3 endometrioid or mucinous or a non-endometrioid tumor.

Tumor AP diameter and tumor/uterine AP diameter ratio showed the best performance for predicting deep MI (AUC of 0.76 and 0.77, respectively). However, when fixing sensitivity at the sensitivity level of subjective assessment (72.3%), the tumor/uterine AP diameter ratio (at a cut-off ≥0.51) had lower specificity (68.9%, i.e., a difference of 7%) and tumor AP diameter (at a cut-off ≥21 mm) had a lower specificity (70.1%, i.e., a difference of 6%) compared to subjective assessment (37).

### 3.4 US techniques for the assessment of cervical involvement

There are very few publications reporting the use of ultrasound to evaluate cervical stromal invasion. From the available data, the results are generally very good for subjective assessment, with sensitivities ranging from 77% to 93% and specificities ranging from 85% to 99% (38, 39).

As previously mentioned, a prospective multicenter study (28) aimed to assess the diagnostic accuracy of subjective and objective ultrasound measurements in the evaluation of cervical invasion. The best objective parameter for the prediction of cervical invasion was the distance from the lower margin of the tumor to the outer cervical os (Dist-OCO), at a cut-off of 20.5 mm. Dist-OCO had a non-significantly higher sensitivity compared with subjective evaluation (73% vs. 54%, *p* = 0.06) but significantly lower specificity (63% vs. 93%, *p* < 0.001), and the AUC did not differ between these methods (28).

Verbakel et al. found that in a study comprising 1,538 patients, the subjective assessment for predicting cervical stromal invasion (CSI) had a sensitivity of 50.7% (95% CI, 42.4%–59.0%) and a specificity of 93.3% (95% CI, 91.8%–94.8%) in patients with a measurable tumor, compared with 49.3% (95% CI, 41.4%–57.3%) and 93.9% (95% CI, 92.6%–95.1%) for the whole IETA-4 cohort. Although Dist-OCO had the best performance for predicting CSI (AUC 0.72), when sensitivity was fixed at the same level as that of subjective assessment (50.7%), Dist-OCO (at a cut-off of ≤18 mm) had a lower specificity (86.5%, i.e., a difference of −7%) than that of subjective assessment (37).

### 3.5 Role of three-dimensional TVUS

Costas et al. performed a systematic review and meta-analysis in order to assess the diagnostic accuracy of 3D-TVUS subjective assessment in the preoperative evaluation of deep myometrial invasion using definitive histology as the reference standard. Nine studies and a total of 581 patients were included (7). The pooled estimated sensitivity, specificity, positive likelihood ratio, and negative likelihood ratio were 84% (95% CI, 73%–90%), 82% (95% CI, 75%–88%), 5 (95% CI, 3.1–7.1), and 0.20 (95% CI, 0.11–0.35), respectively (7).

Another recent systematic review and meta-analysis investigated the diagnostic accuracy of 3D-TVUS and MRI for DMI and cervical invasion, including five studies comprising 450 women. The authors reported a pooled sensitivity, positive likelihood ratio, and negative likelihood ratio of 77% (95% CI, 66%–85%), 4.57, and 0.31, respectively, for detecting DMI using 3D-TVS. The respective values on MRI were 80% (95% CI, 73%–86%), 4.22, and 0.24. For detecting cervical invasion, the pooled ln diagnostic odds ratio was 3.11 (95% CI, 2.09–4.14) for 3D-TVS and 2.36 (95% CI, 0.90–3.83) for MRI. As concluded, 3D-TVUS demonstrated good diagnostic accuracy in terms of sensitivity and specificity for the evaluation of DMI and cervical invasion, with results comparable with those of MRI, thus confirming the potential role of 3D-TVUS in preoperative staging and surgical planning (40).

### 3.6 Inter-observer agreement in TVUS

A study aimed to assess the inter-observer reproducibility among US experts and gynecologists in the prediction by TVUS of deep myometrial and cervical stromal invasion. Sonographic video clips of the uterine corpus and cervix of 53 women with endometrial cancer were integrated into a digitalized survey and evaluated by nine ultrasound experts and nine gynecologists. Findings suggest that preoperative ultrasound staging in EC is best performed by ultrasound experts as they exhibited greater agreement with histopathology and higher inter-observer reproducibility in assessing cervical stroma invasion but not in detecting deep myometrial invasion (4).

### 3.7 TVUS versus MRI performance

MRI is considered the most accurate imaging modality for the preoperative assessment of myometrial and cervical invasion, as well as extra-uterine disease, due to its excellent soft-tissue contrast resolution. The sagittal T2-weighted image delineates the uterine anatomy and is a useful tool in the assessment of the depth of myometrial invasion.

EC typically appears isointense to the myometrium on T1-weighted sequences and hypointense relative to the endometrial lining on T2-weighted sequences. On T1-weighted post-contrast images, the tumoral lesion usually enhances less than the normal myometrium and demonstrates slower enhancement on dynamic contrast imaging (41). The combination of dynamic contrast-enhanced (DCE) and T2-weighted images has reported accuracy values of 98% and 90% for assessing myometrial and cervical invasion, respectively (24). Diffusion-weighted imaging (DWI) is of particular value in patients who cannot receive intravenous gadolinium-based contrast agents and in cases of concurrent adenomyosis (24). On DWI, these lesions typically demonstrate restricted diffusion, appearing as areas of high signal intensity on diffusion-weighted images and of hypo intensity on apparent diffusion coefficient (ADC) maps (42).

In brief, multi-parametric MRI, using a combination of T2-weighted sequences, DWI, and multiphase DCE, stands as the mainstay for imaging assessment of EC (43, 44).

In the last decade, a few studies investigated the competing role of TVUS in the preoperative staging of EC.

A prospective study with 74 consecutively diagnosed cases of EC aimed to compare the accuracy of TVUS and MRI (39). Both techniques performed equally well, with no statistically significant differences. In the assessment of myometrial infiltration, sensitivity, specificity, positive and negative predictive values, and overall diagnostic accuracy were evaluated, revealing values of 84%, 83%, 79%, 88%, and 84%, respectively, for TVUS and 84%, 81%, 77%, 87%, and 82% for MRI, respectively. Regarding the detection of cervical involvement, values of 93%, 92%, 72%, 98%, and 92% were obtained for TVUS and 79%, 87%, 58%, 95%, and 85% for MRI (39).

A recent prospective comparison of the diagnostic accuracies of TVUS and MRI consecutively included 51 women in EC (45). US diagnosed more cases of deep myometrial invasion compared to MRI, however, with no statistical significance. The sensitivity and specificity of TVUS and MRI for myometrial assessment were, respectively, 86% vs 77% and 66% vs. 76%. For the assessment of cervical involvement, both methods correctly diagnosed the same number of cases. The author then concluded similar diagnostic accuracy between the methods (45).

Alcazar et al. published a systematic review and meta-analysis including the results of eight articles, which compared the diagnostic accuracy of TVUS and MRI for detecting myometrial invasion (46). The pooled estimated sensitivity and specificity for the assessment of deep myometrial infiltration were 75% (95% CI = 67%–82%) and 82% (95% CI = 75%–93%) for TVUS and 83% (95% CI = 76%–89%) and 82% (95% CI = 72%–89%) for MRI, respectively, therefore reporting no statistical differences between the methods (*p* = 0.314) (46). A significant heterogeneity was observed for specificity. However, some drawbacks of this systematic review must be pointed out, namely, the small number of papers reported (a total of 8 studies reporting on 560 women, with the number of recruited patients varying from 14 to 177 cases), the time range of those papers varying from 1992 to 2013, and the use of different methodologies, which could explain the reported heterogeneity among the studies.

A head-to-head systematic review and meta-analysis by (47) compared the diagnostic performance of MRI and TVUS for detecting myometrial invasion in patients with low-grade endometrioid EC. The pooled sensitivity and specificity were 65% (95% CI = 54%–75%) and 85% (95% CI = 79%–89%) for MRI and 71% (95% CI = 63%–78%) and 76% (95% CI = 67%–83%) for TVUS, respectively, with no statistical differences between both imaging techniques (*p* > 0.05) (47).

A recent systematic review and meta-analysis by Madár et al. included 18 studies comprising 1,548 patients and performed several subgroup analyses (48). Pooled sensitivity and specificity were, respectively, 76.6% (95% CI, 70.9%–81.4%) and 87.4% (95% CI, 80.6%–92%) for TVUS and 81.1% (95% CI, 74.9%–85.9%) and 83.8% (95% CI, 79.2%–87.5%) for MRI, with no significant differences (sensitivity: *p* = 0.116, specificity: **p** = 0.707). Therefore, the authors indicate comparable diagnostic performance between methods. Notwithstanding, in the subgroup analysis of low-grade EC patients, the specificity of MRI was significantly better (p = 0.044), and a non-significant difference was observed in the no-myometrium infiltration versus myometrium infiltration groups (48).

Although there is scarce evidence comparing the diagnostic yields of two-dimensional TVUS and MRI for detecting cervical infiltration, a recent head-to-head systematic review and meta-analysis by Alcazar et al., including 12 studies and 1,089 patients, revealed no statistical differences when comparing both methods (49). The pooled estimated sensitivity and specificity for diagnosing cervical infiltration were identical for both techniques [69% (95% CI, 51 %–82%) and 93% (95% CI, 90 %–95%) for TVUS and 69% (95% CI, 57 %–79%) and 91% (95% CI, 90 %–95%) for MRI, respectively], with very similar diagnostic performance for diagnosing cervical involvement (49).

As previously shown, a systematic review and meta-analysis compared the diagnostic accuracy of 3D-TVUS and MRI for DMI and cervical invasion (40). With regard to the assessment of DMI, pooled sensitivity, positive likelihood ratio, and negative likelihood ratio were 77% (95% CI, 66%–85%), 4.57, and 0.31, respectively, for 3D-TVUS and 80% (95% CI, 73%–86%), 4.22, and 0.24 for MRI. Bivariate meta-regression showed similar performance of 3D‐TVUS and MRI (*p* = 0.80) for the correct diagnosis of myometrial invasion. Respecting the detection of cervical invasion, the authors reported a pooled diagnostic odds ratio of 3.11 (95% CI, 2.09–4.14) for 3D-TVUS and 2.36 (95% CI, 0.90–3.83) for MRI (40). This work demonstrates the good performance of 3D‐TVUS, thereby reinforcing its value for preoperative staging and surgery planning in patients with EC.

Regarding the inter-observer agreement in MRI, a multicenter retrospective study aimed to perform a multi-reader evaluation using T2-weighted, DWI, and DCE sequences to identify the most accurate sequence and assess its reliability for determining the best protocol. MRI sequences were independently evaluated by four radiologists to identify deep myometrial invasion in a total of 92 patients. The performance of the readers did not show significant differences among DWI, DCE, and the entire protocol, although the latter ensures the highest reliability, particularly for expert readers (82.6%). The highest inter-observer agreement was obtained with the entire protocol by expert readers (intraclass correlation coefficient = 0.77) (50).

### 3.8 TVUS versus frozen section performance

Recent ESGO/ESTRO/ESP guidelines do not recommend the use of the intraoperative frozen section for the assessment of myometrial invasion due to its poor reproducibility and interference with adequate pathological processing (15).

A few studies investigated the role of intraoperative frozen section and evaluated the inter-observer agreement.

A retrospective study published in 2001 evaluated the records of 460 patients with uterine cancer in order to assess the accuracy of intra-operative frozen section (FS) in identifying the features of high-risk uterine disease. The inter-observer reliability was also determined. Tumor grade and depth of myometrial invasion were accurately reported in 88.6% (expected 61.5%, kappa 0.70) and 94.7% (expected 53.8%, kappa 0.89), and error resulting in sub-optimal surgical management occurred in 5.3% of cases (51).

Recently, a retrospective study aimed to assess the role of intraoperative FS in guiding decision-making for the surgical staging of endometrioid EC based on the evaluation of 112 patients (52). The concordance rates of different variables between FS and permanent section were 100%, 89.3% (100/112), 97.3% (109/112), and 95.5% (107/112) for histological subtype, grade, myometrial invasion, and tumor size, respectively. The diagnostic accuracy rate of combined criteria intraoperatively guiding the decision for surgical staging was 95.5% (107/112); the discordance rate of all was 4.5%, resulting in three cases (2.7%) of undertreatment and two cases (1.8%) of overtreatment (52).

Another systematic review and meta-analysis published in 2016 compared the diagnostic performance of intraoperative gross evaluation and intraoperative FS for the assessment of DMI in a total of 35 studies and 6,387 patients (53). Pooled sensitivity and specificity values of 71% and 91% for gross evaluation and 85% and 97% for FS were found, respectively, and both sensitivity (*p* = 0.0008) and specificity (*p* = 0.0021) were significantly higher for FS (53).

A limited body of research has investigated whether preoperative methods, namely, MRI, can safely replace intraoperative frozen sectioning in the local staging of EC.

Many studies still report a slightly better performance of FS for predicting tumor grade (true positive rates of ADC values and FS of 73.3% vs 66.7%, *p* = 0.7; true negative rates of 64.5% vs 98.7%, *p* = 0.01; kappa statistics of 0.23 and 0.73, respectively) (54) and DMI (diagnostic accuracy of 78.8% for MRI versus 81.5% for FS, concordance k = 0.54, *p* < 0.00001) (55) with greater agreement (sensitivity, specificity, positive predictive value, and negative predictive value for DMI on MRI were 57.8%, 92.0%, 69.3%, and 87.5%, with kappa value of 0.53 and 66.7%, 97.9%, 90.9%, and 90.4%, respectively, with a kappa value of 0.71 on FS) (56, 57).

However, some studies considered that both methods are highly accurate (accuracy rates of 88.7% for MRI versus 94.4% in FS, with no statistical differences, *p* = 0.057) (58) and that frozen-section analysis can be avoided if the preoperative MRI study includes DWI sequences and ADC maps (59).

There is scarce evidence comparing TVUS and FS. A comparative prospective study evaluated DMI by TVUS, MRI, and FS, with no statistically significant differences in the overall diagnostic performance for the preoperative and intraoperative assessment of myometrial invasion, although with a slightly better performance for FS (overall accuracy of 78%, 81%, and 91%, and Cohen’s kappa value of 0.534, 0.597, and 0.776, respectively, for TVUS, MRI, and FS) (60).

The same research group has published findings on the evaluation of cervical involvement (59), reaching similar conclusions: no statistically significant differences in the overall diagnostic performance for the preoperative and intraoperative assessment of cervical involvement (61).

### 3.9 Role of intraoperative TVUS

A prospective study by Angeles et al. aimed to evaluate the accuracy of sentinel lymph node (SLN) mapping with a transvaginal ultrasound-guided myometrial injection of the radiotracer (TUMIR) to detect lymph node metastases in patients with intermediate- and high-risk EC, focusing on its performance to detect para-aortic involvement. The authors concluded that the TUMIR method provides valuable information on endometrial drainage in patients at a higher risk of para-aortic lymph node involvement, showing a detection rate of para-aortic SLNs greater than 45% and a high sensitivity and NPV for para-aortic metastases (62). However, further studies on this technique are needed, and given the high detection and accuracy rates of indocyanine green cervical injection (ICG), most protocols are now dismissing use of technetium-99m and endorsing the ICG cervical application site (21).

### 3.10 Artificial intelligence and radiomics in EC

In recent years, there has been a relentless increase in artificial intelligence (AI) applications in the medical domain, aiming to facilitate daily workflows and clinical decision-making. In the gynecologic oncology field, the latest research has validated an AI-driven ultrasound detection of ovarian cancer using a comprehensive dataset of 17,119 ultrasound images from 3,652 patients across 20 centers in eight countries (63).

Radiomics, combining medical imaging (“radio”) with domains such as genomics and proteomics (“omics”), is an emerging quantitative approach to medical imaging, enhancing the existing data available to clinicians by means of advanced and, sometimes, non-intuitive mathematical analysis.

A multicenter, retrospective, observational study by Moro et al. aimed to develop and validate radiomics models applied to ultrasound images to differentiate high-risk and low-risk EC. Radiomics, clinical-ultrasound-based, and mixed models were developed to distinguish between high- and low-risk groups. The authors showed that radiomics seems to have some ability to distinguish low-risk EC and a better ability to distinguish high-risk EC, but the addition of radiomics features to clinical-ultrasound models did not improve the model’s performance (64).

The adoption of this innovative tool may impact screening, diagnosis, and prognosis. An ongoing prospective trial intending to compare data obtained from radiomic analysis and molecular/genomic profiling is currently underway. Given the costs and turnaround time associated with the implementation of molecular testing, the rationale for this research is to validate radiomics applied to ultrasonographic images as an effective, innovative, and inexpensive method for tailoring operative and postoperative treatment in EC (65).

Additional research on radiomics is required to validate the performance of these models and evaluate their potential role in clinical practice.

## 4 Conclusion

Preoperative imaging is crucial to enable a tailored surgical procedure in endometrial cancer, and TVUS and pelvic MRI are the preferred methods for the local staging.

TVUS is as a promising tool in the preoperative work-up of endometrial carcinoma. It is a low-cost and promptly accessible method, with no harm or discomfort to the patient, allowing a dynamic evaluation of the pelvic structures and the anatomical relation to the surrounding viscera, which presents a clear advantage over the remaining preoperative techniques. Additionally, it is performed preoperatively, allowing a tailored surgical procedure and avoiding the increase in operative times and unnecessary costs inherent to intraoperative frozen section.

Preoperative TVUS performed by experts seems to provide comparable diagnostic yields for determining myometrial invasion in EC compared to other preoperative or intraoperative tools, with no significant differences between methods in the most recent systematic reviews and meta-analyses.

Regarding cervical stromal involvement, the latest head-to-head systematic review and meta-analyses found no statistical differences between both methods, indicating very similar diagnostic performance for diagnosing cervical involvement.

However, all imaging methods are restrained by non-perfect performances and limitations in reproducibility. Comparable diagnostic inaccuracy reflects the same drawbacks in both methods, revealing a similar tendency to overestimate myometrial invasion and underestimate cervical stromal invasion.

Agreement between methods is reasonable, suggesting that the best alternative will be highly dependent on the availability and expertise of each institution. Because of imaging availability, ultrasound by experts will become the preferred option, whereas MRI will remain relevant in cases of reduced acoustic visibility due to limiting factors such as uterine pathology, acoustic shadows, or uterine position.
